# Successful catheter cryoablation for premature ventricular contractions originating from the para‐Hisian region

**DOI:** 10.1002/ccr3.2246

**Published:** 2019-06-25

**Authors:** George Suzuki, Akihiko Yotsukura, Tadafumi Nanbu, Masayuki Sakurai

**Affiliations:** ^1^ Division of Arrhythmias and Cardiac Electrophysiology, Department of Cardiovascular Medicine Hokko Memorial Hospital Sapporo Japan

**Keywords:** catheter cryoablation, para‐Hisian origin, premature ventricular contractions

## Abstract

We achieved successful catheter cryoablation in a patient with para‐Hisian premature ventricular contractions (PVCs) without conduction disturbance using the freeze‐thaw‐freeze method while observing the atrial‐His bundle interval. Cryoablation could be considered an alternative to radiofrequency ablation for patients with para‐Hisian PVCs.

## 1 INTRODUCTION

While the para‐Hisian region is not necessarily a rare source of idiopathic monomorphic ventricular arrhythmias,^1^, ^2^, ^3^, ^4^ catheter ablation for this region carries a risk of inadvertent atrioventricular conduction disturbance. Successful radiofrequency catheter ablation of para‐Hisian ventricular arrhythmias has been reported.^5^, ^6^, ^7^, ^8^ However, reports of cryoablation for this region have been limited. Herein, we report a case of successful catheter cryoablation of para‐Hisian idiopathic premature ventricular contractions (PVCs) without any complications.

## 2 CASE REPORT

**Figure 1:**
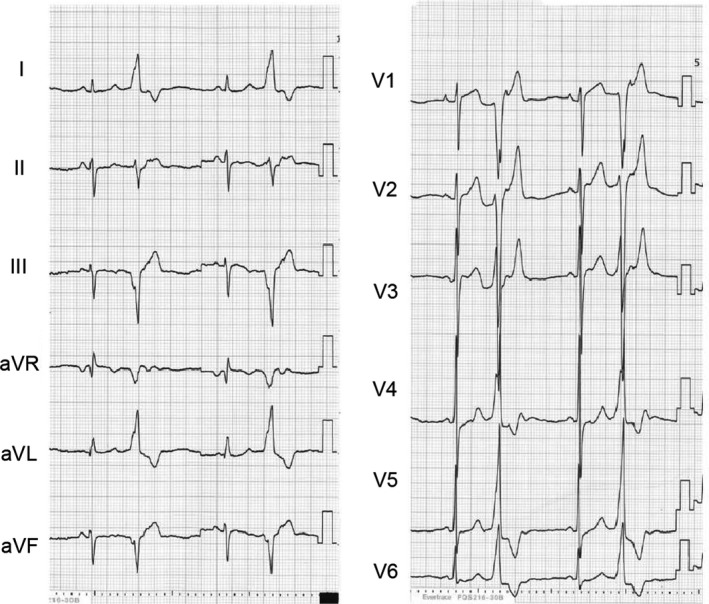
The 12‐lead superficial ECG obtained in the outpatient department suggests that the PVCs originated near the His bundle in the right ventricular septum. ECG, electrocardiography; PVC, premature ventricular contractions

A 76‐year‐old woman experienced nocturnal dyspnea and obviously fewer pulses than usual for over a year. Her primary physician detected frequent PVCs, which prompted her referral to our outpatient department. Neither cardiac computed tomography nor echocardiography showed structural heart disease. A Holter monitor recorded PVCs for 35.1% in 24 hours. The 12‐lead electrocardiography (ECG) showed tall R waves in I and aVL, in addition to a near‐QS pattern in V1, relatively narrow QRS waves in V1‐2, and a precordial transition zone between V3 and V4 (Figure 1). All these findings strongly suggested that the PVCs originated from the right ventricular septum close to the His bundle.^1^, ^2^, ^4^, ^9^, ^10^, ^11^

**Figure 2:**
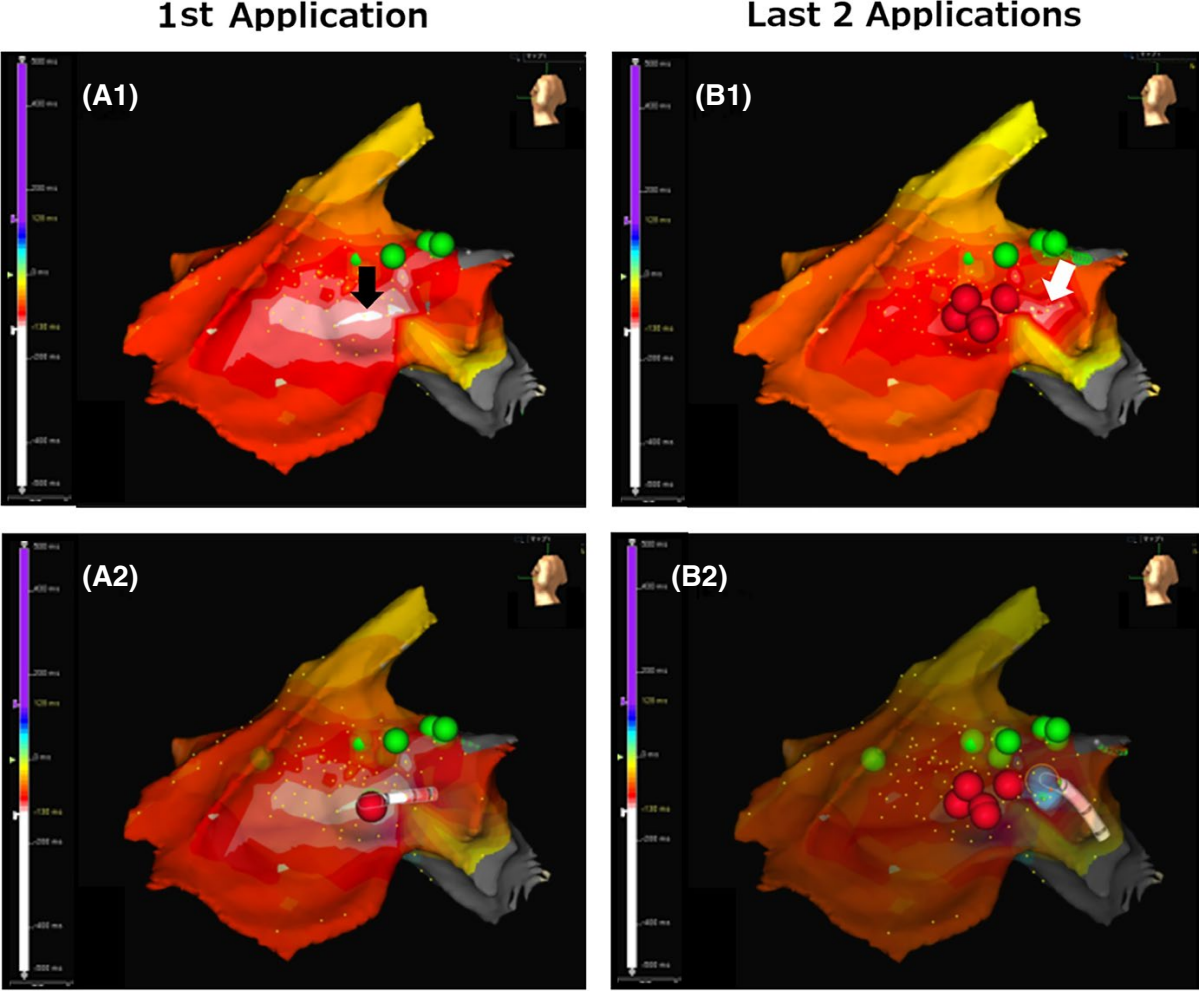
The earliest activation site by three‐dimensional mapping and cryoablation sites. Green tags indicate the points where the His potential was recorded with a hexapolar diagnostic catheter and/or a cryocatheter. A1: The activation map initially created before the first application of cryoenergy. Black arrow indicates the earliest activation site of PVCs. A2: Applications shown by red tags were unsuccessful. B1: Map modified using newly acquired potentials superimposed on the initially created map. The earliest activation site shifted to a higher position (white arrow). B2: Two blue tags indicate the sites at which cryoablation was clearly effective. Only a 5‐mm gap exists between the His potential and the cryocatheter tip. The last two application sites are indicated by blue tags. PVC, premature ventricular contractions

**Figure 3:**
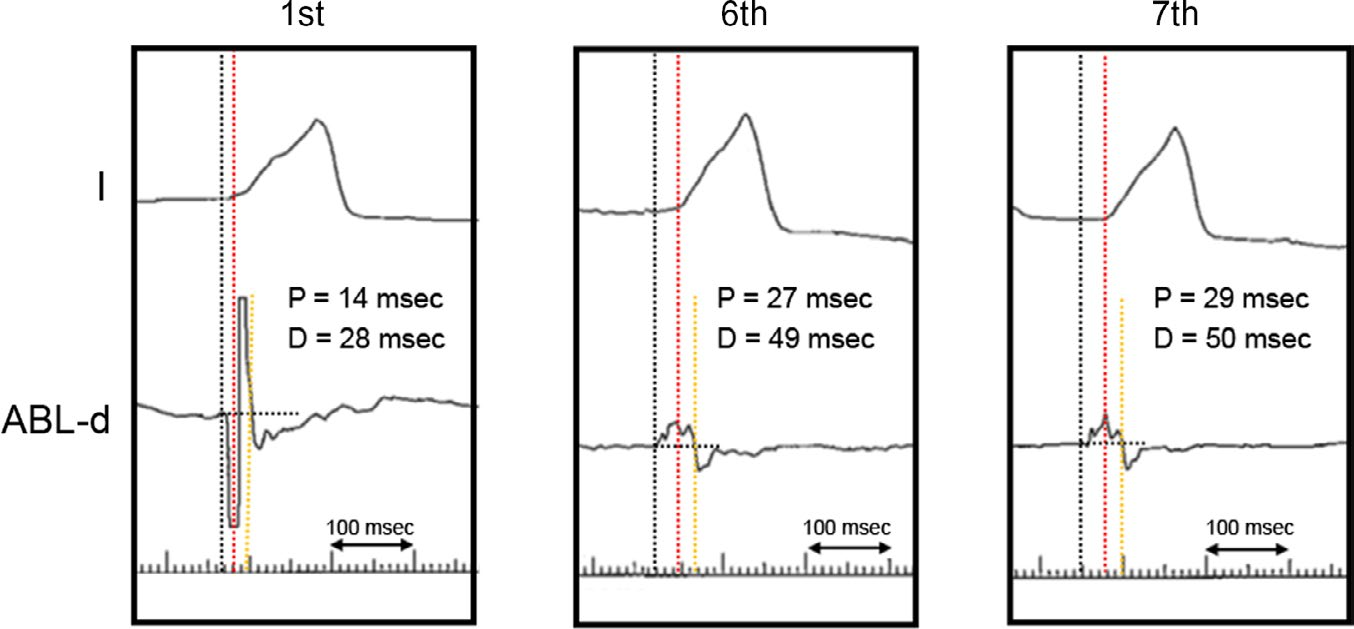
The precedence of the distal bipolar electrogram from the rise of QRS complexes and the R‐wave duration during the clinical PVCs at the earliest activation site. The precedence at the last two cryoablation sites is significantly greater than that at the first ablation site. In addition, the R‐wave durations at the last two ablation sites are significantly longer and possess higher frequencies. Black and yellow dotted lines represent the rise, and terminal point of the R‐wave recorded by the distal bipolar electrogram, respectively. The red dotted line represents the rise of the QRS complex recorded by the surface ECG. D, Duration; ECG, electrocardiography; P, precedence; PVC, premature ventricular contractions

**Figure 4:**
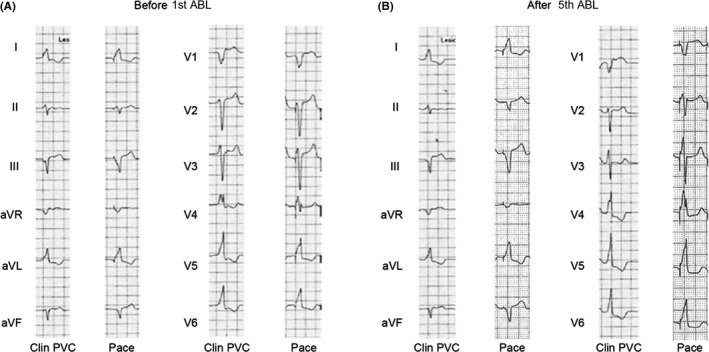
A: Comparison between clinical PVCs and the pace map recorded just prior to the first application of cryoenergy. A good pace map was obtained. B: Comparison between clinical PVCs and the pace map recorded after the fifth cryoablation attempt. The precordial transition zone shifted leftward. The sixth attempt had no effect on clinical PVC morphology. ABL, ablation catheter; Clin PVC, clinical premature ventricular contraction; PM, pace mapping

**Figure 5:**
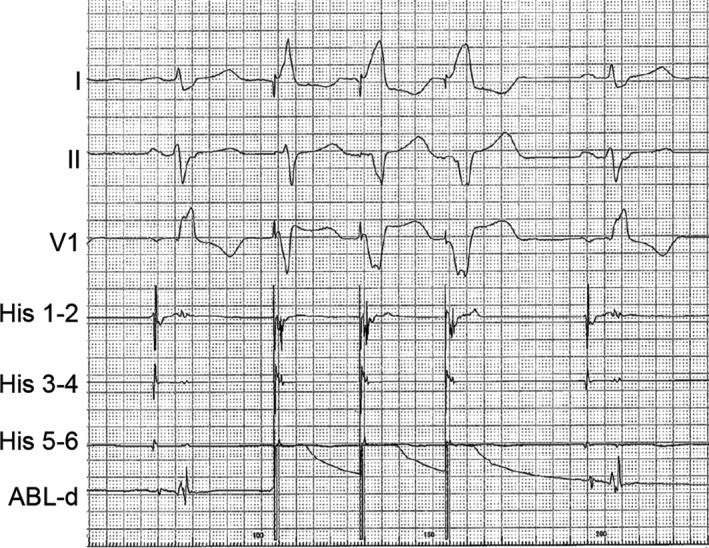
Pacing with the maximum output at the earliest activation site just before the sixth attempt. The first beat became narrow, which suggested that the pacing captured the His bundle

After informed consent was obtained, a cardiac electrophysiological study was performed with the patient fasting and not taking antiarrhythmic drugs. A 4‐Fr hexapolar electrode catheter (Supreme^™^, St. Jude Medical, Inc) was positioned in the His bundle region. A 7‐Fr straight eicosapolar electrode catheter (CristaCath^®^, Biosense Webster, Inc) was carried into the right ventricle via an 8.5‐Fr bidirectionally deflectable sheath (Agilis^™^ NxT, St. Jude Medical, Inc), and a three‐dimensional (3D) electroanatomical map was created using EnSite^™^ NavX^™^ System (St. Jude Medical, Inc). The His potential location was marked on the 3D map. A total of 193 points of potentials were acquired. The mean amplitude of five consecutive His potentials recorded with the His catheter was 0.10 mV. The activation map revealed that the earliest ventricular activation of PVCs was located 5 mm inferior to the His bundle (Figure 2A1), where a 6‐mm‐tip‐cryoablation catheter (Freezer^™^ Xtra, Medtronic CryoCath LP) was subsequently positioned (Figure 2A2). The PVC potentials recorded by the distal electrode of the ablation catheter preceded the rise of the QRS on surface ECG by 14 msec (Figure 3). A good pace map was thereby obtained (Figure 4A). Two sequential cycles of cryoenergy were delivered at −80°C for 4 minutes while monitoring the PR interval with a few seconds of interruption for thawing.^12^ The first ablation attempt only minimally decreased the number of PVCs. Another four applications of cryoenergy were placed at an adjacent region, but the only effect was to rotate the QRS transitional zone counterclockwise (Figure 4B). Therefore, the newly acquired five points of potential were superimposed on the existing activation map. The earliest activation site shifted to a higher position on the revised activation map (Figure 2B1). The distance between this activation site and the first targeted site was <5 mm. Therein, the pace mapping did not match the clinical PVCs well (Figure 4B). However, the potential recorded at the cryocatheter tip preceded the rise of the QRS complex by 27 msec, which was much earlier than that obtained at prior attempt sites (Figure 3). Compared with the previously targeted points, the bipolar electrogram recorded by the cryocatheter showed a significantly longer duration (49 msec) and a higher frequency potential (Figure 3).^13^ Prior to delivering cryoenergy, we made sure that the His potential was not recorded with the distal electrode of the cryocatheter. However, pacing at the site with maximum output (9.9 V) made the QRS width slightly narrow in the first beat, suggesting that the first pacing pulse was captured at the His bundle (Figure 5). The sixth attempt eliminated the PVCs within 10 seconds after the start of the application (Figure 2B2). Nevertheless, the PVCs appeared again soon after the discontinuation of cryoenergy. The potential recorded at the site adjacent to the sixth site further preceded the QRS complex by 29 msec (Figure 2B2, Figure 3). The PVCs were completely abolished within 21 seconds after cryoenergy application. The last two attempts were also performed with the freeze‐thaw‐freeze method (delivered at −80°C for 4 minutes, interrupted only for a few seconds for thawing), similar to previous sequential attempts.^12^ The total procedure and fluoroscopy times were acceptable (86 minutes and 17 minutes, respectively). Neither junctional rhythm nor ventricular ectopy was induced while delivering cryoenergy. At the completion of all procedures, neither atrial‐His bundle (AH) nor HV intervals showed any change compared to baseline values.

A 24‐hours Holter monitor placed on the patient, and 2 days after, the catheter procedure only recorded 15 isolated PVCs. The patient has been symptom‐free since the procedure. The 12‐lead ECG showed no PVCs 12 months after cryoablation.

## 3 DISCUSSION

Frequent para‐Hisian PVCs were safely abolished with catheter cryoablation. Ventricular arrhythmias arising from this region are not common, but also are not necessarily rare.^1^, ^2^, ^4^ Some previous reports indicated that radiofrequency catheter ablation is less effective and results in a higher recurrence rate for ventricular arrhythmias originating from the near‐His region than for those from other sites in the right ventricle.^2^, ^3^ This may be the result of reluctance to perform aggressive ablation for fear of atrioventricular conduction disturbances. Cryoenergy has several desirable characteristics for use in regions susceptible to atrioventricular conduction disturbances associated with radiofrequency ablation.^14^, ^15^ Nevertheless, little has been reported on the feasibility of catheter cryoablation for para‐Hisian ventricular arrhythmias. To our knowledge, only one report, which was recently published from the Mayo Clinic, described 10 patients with para‐Hisian ventricular arrhythmias treated with catheter cryoablation.^16^ In their study, 8 of 10 patients had received radiofrequency ablation, resulting in failure before cryoablation. The other two patients who did not receive radiofrequency ablation were successfully treated with catheter cryoablation. However, their report did not describe their definition of para‐Hisian region nor concretely show how close it was from the origins to the His bundle. In the current case, we demonstrated the safety and efficacy of using catheter cryoablation alone in a patient with para‐Hisian PVCs, clearly showing the proximity of the para‐Hisian region to the His bundle.

Among the eight cases which previously resulted in failure of using radiofrequency ablation, five were successfully treated with catheter cryoablation. The ideal characteristics of cryocatheter ablation for patients with para‐Hisian PVCs are as follows:

First, the cryocatheter tip adheres to the myocardium and is unaffected by the heartbeat, and the target site will remain frozen once the tip is in the right position. This characteristic is especially valuable in patients whose arrhythmia originates at a site wherein retaining the position of the ablation catheter is difficult.

Second, cryoenergy creates more homogeneous and clearly circumscribed lesions compared with radiofrequency; therefore, cryolesions affect less surface area. Conversely, the depth of lesions created by both are comparable. These cryoablation properties produce a safer procedure by limiting injury to the true target region, especially when critical regions are affected, as in the present case.

Third, the most important basis for safety of cryoablation is reversibility, which is never available with radiofrequency ablation. Hence, irreversible conduction disturbances will not occur as long as ablation is discontinued as soon as the AH interval begins to lengthen.

Nevertheless, even with cryocatheter ablation, we still need to be aware of conduction disturbances while ablating near‐His bundle regions. As described above, Miyamoto et al^16^ recently reported 10 patients with para‐Hisian PVCs treated with cryocatheter ablation. Cryoablation could not be carried out at the actual focus in two patients because of transient atrioventricular blocks. One of the seven patients, in whom cryoablation was carried out, developed a complete atrioventricular block during cryoenergy application, which then required implantation of a permanent pacemaker. In their study, most of their patients had previously undergone unsuccessful radiofrequency ablations, which might have been the basis for this complication. Additionally, the patient who underwent pacemaker implantation already had a first‐degree atrioventricular block before cryoablation. However, their report issues a warning. Even if conduction disturbance is not observed during cryomapping, there is no guarantee that it will not occur during cryoablation with a target cooling range.^17^ Furthermore, in the target temperature range of −60°C to −80°C, subcellular organelle structures are destroyed within 1 minutes followed by irreversible cellular death.^18^ Regardless of whether one chooses radiofrequency or cryoablation, to avoid conduction disturbances, we never ablate the site at which His potentials are recorded with the distal electrode of the ablation catheter. We followed this policy in the present case as well, as we always do.

In accordance with a good pace map at the earliest site in the primary 3D activation map, we delivered cryoenergy there for the first attempt, but it only affected the morphology of the QRS complex, and the number of PVCs barely decreased. Conversely, at the earliest site in the latterly superimposed 3D map, pace mapping did not match clinical PVCs, while we still attempted cryoablation therein with trust in the precedence, R‐wave duration, and frequency of the local potential, resulting in complete elimination of PVCs. This discrepancy should be explained with the existence of preferential conductions. The origin must have been in the last two targeted sites, and the first targeted site could have been one of the exits. Moreover, the exit could shift to another site by ablating the first targeted site, which could change the QRS morphology and QRS transition zone. Particularly, in the setting of fewer PVCs available during electrophysiological study, pace mapping can be more reliable than activation mapping. However, the present case reminds us that we should always evaluate the local potentials of each site with greatest care before applying energy to avoid unnecessary ablation, because pace mapping has several pitfalls, such as the issue described above.^19^

While the reversibility provides safety, it might also indicate that achieving a permanent and complete cellular change with cryoenergy is less promising compared with that with radiofrequency ablation. Some studies indicate that the recurrence rate in cryoablation was higher than that in radiofrequency ablation in patients with atrioventricular nodal re‐entrant tachycardia.^20^, ^21^ However, none of those studies were limited to patients with slow pathways critically close to the His bundle. With studies limited to such cases, more aggressive ablation can be done as there was a low risk of conduction disturbance, which may overcome the inferiority of tissue change permanently in cryoablation in contrast to radiofrequency ablation.

## 4 CONCLUSION

We performed catheter cryoablation of PVCs, originating from the para‐Hisian region, without causing atrioventricular conduction disturbances. This desirable outcome, which has persisted for a year, results from the optimal use of cryoenergy characteristics and the use of a cryocatheter. Thus, catheter cryoablation could be considered an alternative to radiofrequency ablation to treat PVCs originating from the para‐Hisian region.

## CONFLICT OF INTEREST

None declared.

## AUTHOR CONTRIBUTIONS

GS: interpreted the clinical data and results in the present case and substantially wrote the initial draft of the manuscript. AY, TN, and MS: interpreted the data and assisted in the preparation of the manuscript. All authors critically reviewed and revised the manuscript. All authors discussed and approved the final version of the manuscript.
